# Measurement of Completeness and Timeliness of Linked Electronic Health Record Pharmacy Data for Early Detection of Nonadherence to Breast Cancer Adjuvant Endocrine Therapy

**DOI:** 10.1200/CCI.24.00115

**Published:** 2024-12-12

**Authors:** Chelsea McPeek, Shirlene Paul, Jordan Lieberenz, Mia Levy

**Affiliations:** ^1^ RUSH University Cancer Center, Chicago, IL; ^2^ Division of Hematology, Oncology and Stem Cell Transplant, Department of Medicine, RUSH University Medical Center, Chicago, IL

## Abstract

**PURPOSE:**

This retrospective cohort study evaluated whether linked electronic health record (EHR) pharmacy data were adequately complete and timely to detect primary nonadherence to breast cancer adjuvant endocrine therapy (AET).

**MATERIALS AND METHODS:**

Linked EHR pharmacy data were extracted from the EHR for patients with stage 0 to III breast cancer who had their first prescription order for AET between 2016 and 2021. Patients with the first dispense event within 90 days of the prescription were classified as having sufficient or insufficient data available for early detection of primary adherence.

**RESULTS:**

A total of 1,446 eligible patients had a first AET prescription order between 2016 and 2021; these orders were routed to 871 unique pharmacies, of which 856 (98.2%) were contracted with the linked EHR pharmacy database and 15 (1.8%) were not contracted. Among the 1,428 patients with a first prescription sent to a contract pharmacy, 164 (13%) had incomplete linked EHR pharmacy data refresh events to assess primary adherence. Among the 1,244 patients with at least 1 refresh event after their first prescription, 82% occurred within 90 days and were sufficiently timely for early detection of primary adherence. Overall, 32% of patients would benefit from an intervention to verify or improve primary adherence to AET.

**CONCLUSION:**

Although linked EHR pharmacy data have adequate completeness of contract pharmacy data, local configurations of data refresh events tailored to medication reconciliation workflows are incomplete (13%) and insufficiently timely (32%) to fully support clinical decision support (CDS) for early detection of primary medication nonadherence. Prospective CDS interventions using linked EHR pharmacy data are possible with enhancements to the frequency and timeliness of refresh events.

## BACKGROUND AND SIGNIFICANCE

Adjuvant endocrine therapy (AET) is a class of oral anticancer drugs that improves long-term survival in women with stage I-III hormone receptor–positive disease when taken for at least 5 years^1-7^ and up to 10 years^8,9^ and is considered the standard of care.^10,11^ Despite the proven clinical benefit of AET, many breast cancer survivors either fail to fill their first prescription (primary nonadherence), fail to take the correct dosage at the prescribed frequency (nonadherence), or discontinue therapy (nonpersistence). A systematic review of 29 studies showed the prevalence of adherence ranging from 41% to 72% and discontinuation (ie, nonpersistence) ranging from 31% to 73%, measured at the end of 5 years of treatment.^12^ Patients with nonadherence or nonpersistence to AET have an increased risk of death from breast cancer.^13^ However, these studies typically measure adherence and persistence in the population of patients who filled their first prescription and do not include patients who never filled their first prescription.

**CONTEXT**

**Key Objective**
In patients with breast cancer who are prescribed adjuvant endocrine therapy (AET), is the quality of linked electronic health record (EHR) pharmacy data adequate to detect whether the prescription was filled within 90 days of the order (*primary adherence*)?
**Knowledge Generated**
Only 68% of the patient cohort had documented primary adherence according to the linked EHR pharmacy data. The remaining 32% of patients had either incomplete data (14%) or lack of AET dispense (*primary nonadherence*) within 90 days (18%).
**Relevance *(J.L. Warner)***
This study illustrates the importance of careful evaluation of multimodal data sources, and is a strong word of caution for anyone relying exclusively on EHR data for cancer drug exposure research. Standardized approaches for medication data refreshes are needed.*
*Relevance section written by *JCO CCI* Editor-in-Chief Jeremy L. Warner, MD, MS, FAMIA, FASCO.

Unfortunately, oncologists are not routinely notified when patients do not fill or refill their prescriptions at the prescribed frequency. Typically, oncologists rely on the patient to report if they filled their prescription and took the medication as directed within the proposed time frame. The lack of a real-time closed-loop ordering and dispensing system in the ambulatory setting is a missed opportunity for early intervention to address primary medication nonadherence.

Linked electronic health record (EHR) pharmacy data provide medication dispensing tracking information in the EHR obtained directly from pharmacies through linked EHR pharmacy databases. Linked EHR pharmacy data could close the communication gap between pharmacies, clinicians, and patients by providing a route to receive medication dispensing tracking information in the EHR.^14^ However, to determine the feasibility of using linked EHR pharmacy data to effectively identify primary nonadherence, we must evaluate the completeness and timeliness of linked EHR pharmacy data to EHR data interface transactions.

Linked EHR pharmacy data were initially designed to facilitate the medication reconciliation process, as encouraged by the Centers for Medicare and Medicaid Services EHR Incentive Program.^15^ The core objectives of the initial stages of the program encouraged clinicians to submit prescriptions electronically using EHR technology and to perform medication reconciliation at relevant clinical encounters.^16,17^ As such, the triggering event for refreshing the linked EHR pharmacy data was focused on when a clinical encounter would occur. Without a clinical encounter, the linked EHR pharmacy data were not always up to date.

**FIG 1.**
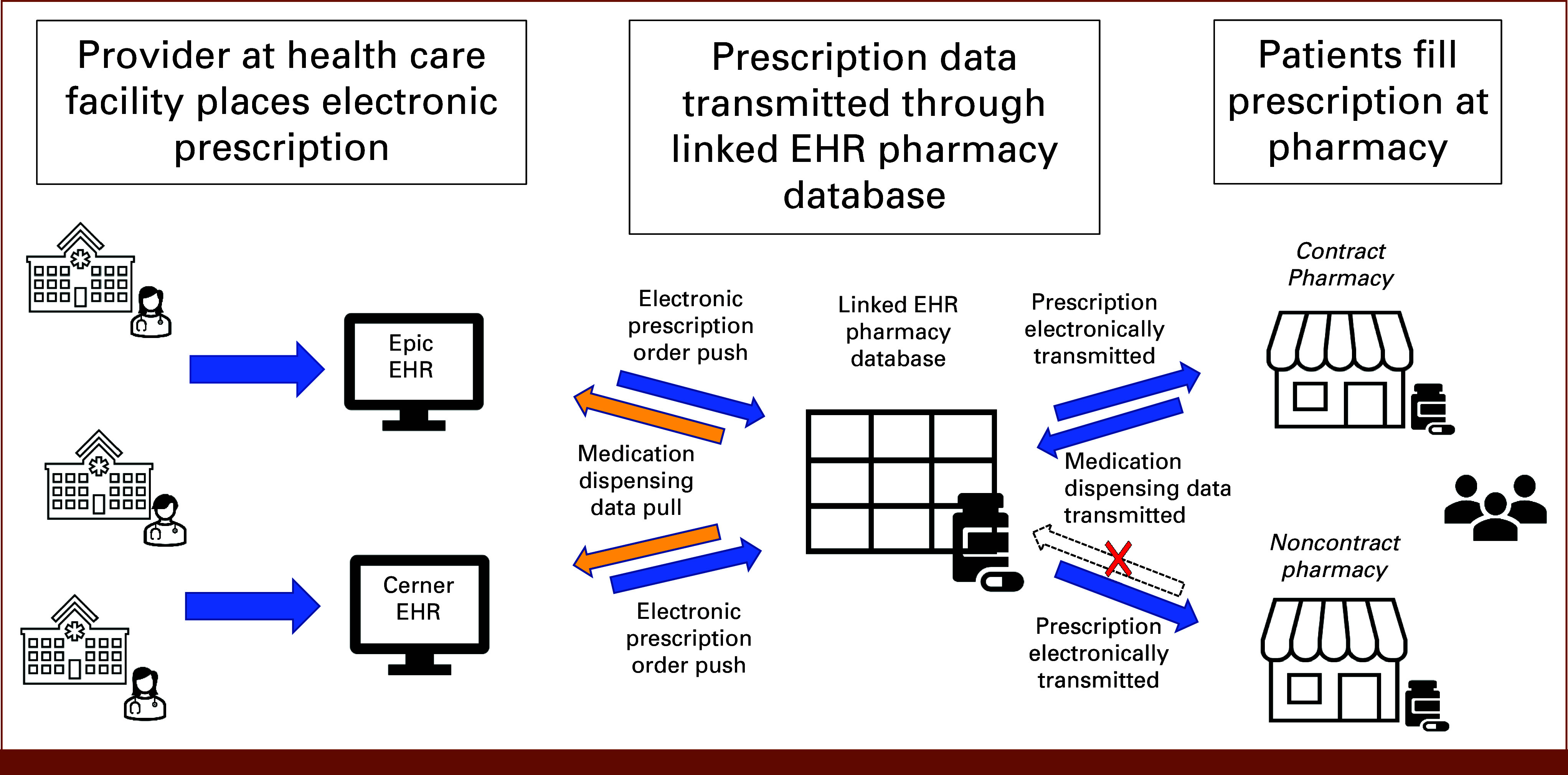
Data flow between EHR, linked EHR pharmacy database, and pharmacy. EHR, electronic health record.

Linked EHR pharmacy databases can improve medication reconciliation activities and could be used to leverage pharmacy data for clinical decision support (CDS). The pharmacy data flow from the EHR e-prescribing system(s) through the linked EHR pharmacy database and downstream to the pharmacy (Fig 1). The pharmacy dispensing data are then transmitted back through the linked EHR pharmacy database. Institutions can request a refresh of patient-specific dispensing information on demand for a transaction fee paid to the linked EHR pharmacy database. This system, however, is not without limitations.

A linked EHR pharmacy database does not contract with all ambulatory pharmacies, resulting in *incomplete* dispensing data (Fig 1, arrow with X). Furthermore, clinical systems must request a data refresh for individual patient dispense data rather than receiving pushed data when the medication is dispensed (Fig 1, yellow arrows, medication dispensing data pull). Such systems are not actual real-time closed-loop ordering and dispensing systems as configured in the inpatient setting. Health care delivery systems can configure their ambulatory EHR to trigger a data refresh event. However, they must balance the frequency of refresh events on the basis of clinical need and per-transaction costs at the enterprise level. These factors may limit the *timeliness* of linked EHR pharmacy data for certain CDS use cases, such as medication adherence.

Patients with breast cancer provide an ideal use case to measure the impact that linked EHR pharmacy data can have on early detection of primary medication nonadherence. In this study, we seek to evaluate the completeness and timeliness of linked EHR pharmacy data for early detection of primary medication nonadherence events for patients with breast cancer who do not fill their first AET prescription within 90 days.

## MATERIALS AND METHODS

Deidentified data for this retrospective cohort study were extracted from the Epic EHR of an urban academic medical center with hospitals and clinics in the Chicago metropolitan area. This study was deemed exempt by the institutional review board (#19121001-IRB01) and was granted a waiver of consent. This study was compliant with the Health Insurance Portability and Accountability Act.

Patients with a breast cancer International Classification of Diseases (ICD) 10 billing code were included if they did not have metastatic disease documented in the cancer staging forms and they had a first prescription order from a breast medical oncology clinician at the study institution for one of the four AET drugs (tamoxifen, anastrozole, letrozole, exemestane) between 2016 and 2021. Patients with ductal carcinoma in situ (stage 0) were included. Patients were excluded if they had documented metastatic disease or if they lacked cancer staging data. This study did not distinguish between patients receiving endocrine therapy in the neoadjuvant, adjuvant, or recurrence setting so long as they did not have documented metastatic disease and it was their first prescription for endocrine therapy at the study institution.

The health system contracts with the commercial linked EHR pharmacy database, Surescripts, to integrate ambulatory pharmacy dispensing data into the local Epic EHR. The health system has defined a set of clinical encounters that trigger a data refresh event; the system requests an update of ambulatory dispensing data for an individual patient over the previous 365 days. In this study, a data refresh event is triggered by various encounters such as ambulatory appointment encounters, telephone encounters, emergency department visits, and hospital admissions. If a patient does not have another qualifying encounter within 365 days after an initial prescription order, the system has no way of knowing whether the medication was ever filled.

The medication history provided by the linked EHR pharmacy database contains three specific dates: written (order) date, last filled date (when the pharmacy fills the medication, which is typically within 24 hours even if the patient does not pick it up), and sold (patient pickup) date. When available, the sold date is used as the dispense date, but if it is not provided, the last prescription filled date is often used as a proxy for the sold date. At the study institution, if the patient does not pick up the prescription within a reasonable amount of time (ie, 14 days), the dispense date would then change and the entire claim would be reversed from insurance. Because of the relatively short amount of time where this would be corrected, it would not affect estimates of primary adherence.

Previous studies have used varying definitions of primary adherence, ranging from days to several months; a standardized definition has not been established.^18,19^ For this study, primary adherence to AET was defined as when the prescription was filled within 90 days of the first prescription order. This time point was selected for the study as it is clinically reasonable for AET.

The cohort was classified into having insufficient data or sufficient data for the assessment of primary adherence on the basis of prescription ordering, filling, and linked EHR pharmacy data refresh dates. Patients whose first prescription was sent to a pharmacy that does not contract with the linked EHR pharmacy database were classified as having insufficient data available to assess primary adherence. Similarly, patients who were lost to follow-up and never had another qualifying encounter to trigger a linked EHR pharmacy data refresh event were classified as having insufficient data available to assess primary adherence. For example, a patient who initially came to the institution and received an AET prescription order but then opted to receive their care elsewhere and did not return to the study institution would have insufficient data to assess primary adherence.

Patients were classified as having sufficient data to evaluate primary adherence if there was at least 1 linked EHR pharmacy dispense refresh event within 365 days of their first AET order. Among patients with sufficient data to assess primary adherence, those with evidence of an AET dispense event within 90 days of their first AET prescription order were classified as having primary adherence to AET.

**FIG 2.**
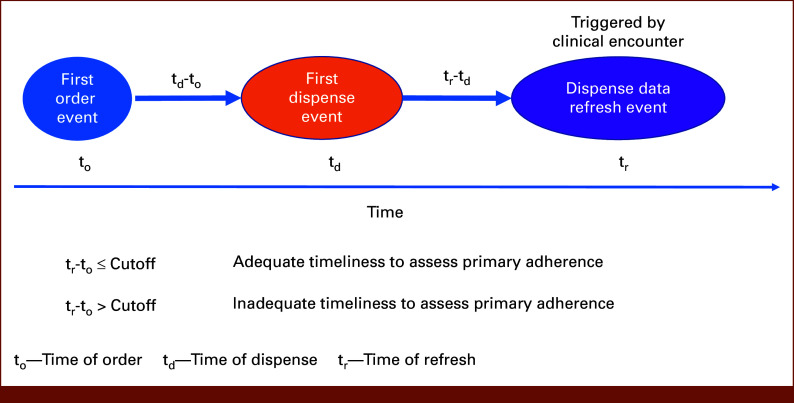
Method of classifying timeliness to assess primary adherence.

If the care team was to intervene for patients who did not fill their initial prescription within 90 days, the timeliness of the linked EHR pharmacy data refresh event would be essential for early detection of primary nonadherence. Figure 2 describes the method for classifying timeliness on the basis of the dates of the prescription order, dispense event, and refresh event. In this study, we allowed up to 365 days from the first AET prescription order for a dispense event and a refresh event to occur. A cutoff of 365 days was selected as medication prescriptions are valid for 1 year. Furthermore, we defined a refresh event within the first 90 days of the first AET prescription order as adequate early detection, whereas a refresh event past that period was defined as inadequate early detection to determine whether a patient was primarily adherent.

A patient who has an order and a refresh event within the cutoff period is considered to have adequate timeliness to assess primary adherence, whereas a patient who does not have the two events within the cutoff period is considered to have inadequate timeliness to assess primary adherence. A patient who has an order and a dispense event within the cutoff period but does not have the refresh event within this time is considered to have inadequate timeliness to assess primary adherence.

## RESULTS

### Eligibility Determination

**FIG A1.**
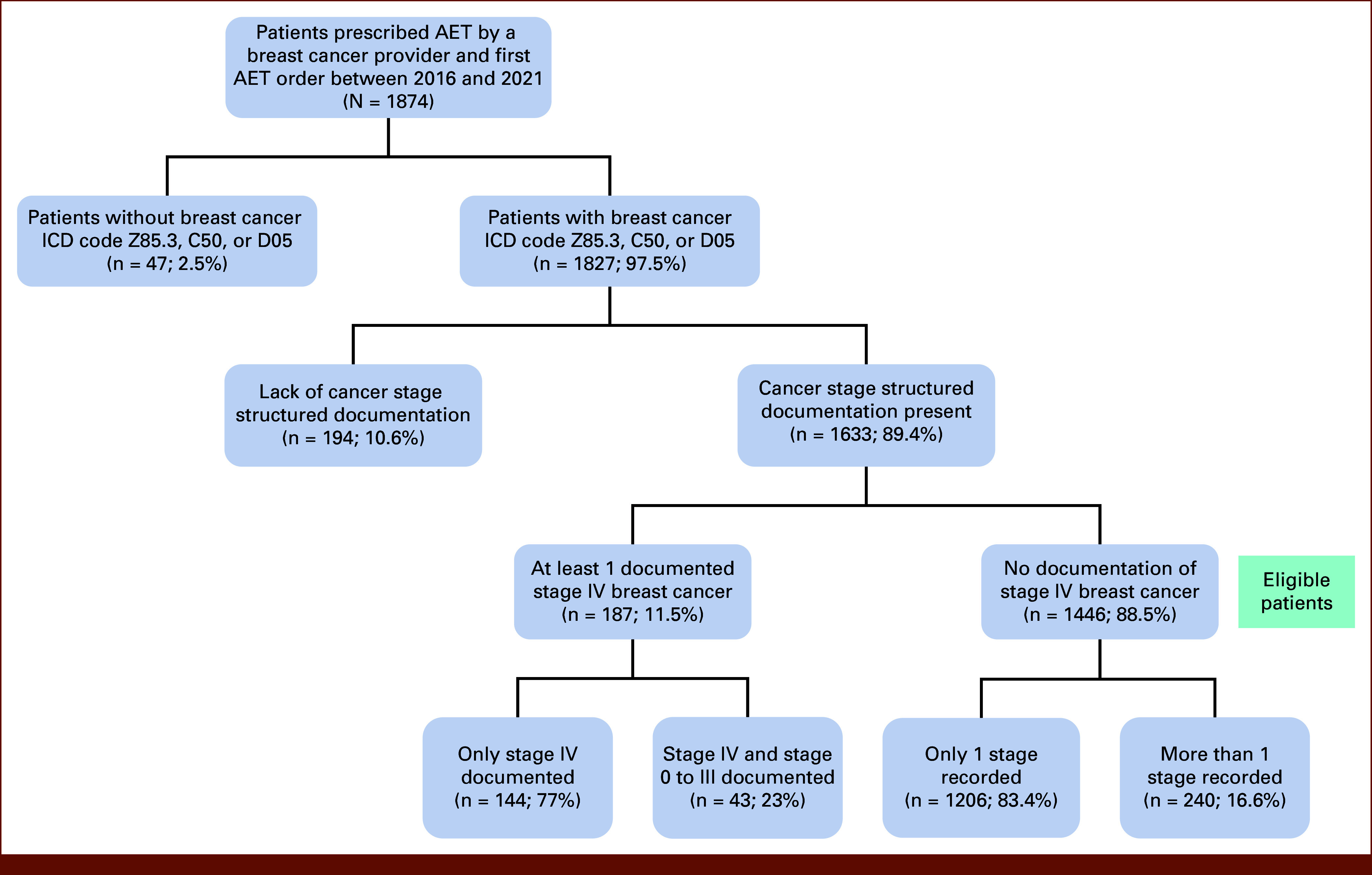
CONSORT flow diagram of the eligible study population. AET, adjuvant endocrine therapy; ICD, International Classification of Diseases.

As shown in Appendix Fig A1, 1,874 patients were prescribed one of the four endocrine therapy drugs by qualifying clinicians between January 1, 2016, and December 31, 2021. Of these, 1,827 (97.5%) patients had a documented breast cancer ICD-10 billing code. Because of a lack of structured cancer staging data, 194 (10.6%) patients were excluded. Patients categorized as Stage Group Unknown (218 [15%]) did not have a stage group documented (0, 1, 2, or 3), most likely because they had neoadjuvant therapy, but they did have M status documented, which was enough to determine eligibility. Among the staged patients, 1,446 (88.5%) patients had no evidence of metastatic disease; these patients were eligible for our study and made up the study cohort. Table 1 shows the study cohort's demographic information, including sex, race, age, and distribution of breast cancer staging among patients.

**TABLE 1.** Demographics and Breast Cancer Stage Distribution of Study Population

Demographic Category	Population (N = 1,446)	Percentage
Female	1,438	99.4
Male	8	0.6
Age, years (median, range, IQR)	62 (24-96; 18)	
Race		
White	780	53.9
Black or African American	395	27.3
Hispanic or Latino	166	11.5
Asian	50	3.5
Other	48	3.3
American Indian or Alaska Native	7	0.5
Stage		
Stage 0	86	6
Stage I	804	56
Stage II	241	16
Stage III	97	7
Stage group unknown (M0)	218	15

### Primary Medication Adherence

**FIG 3.**
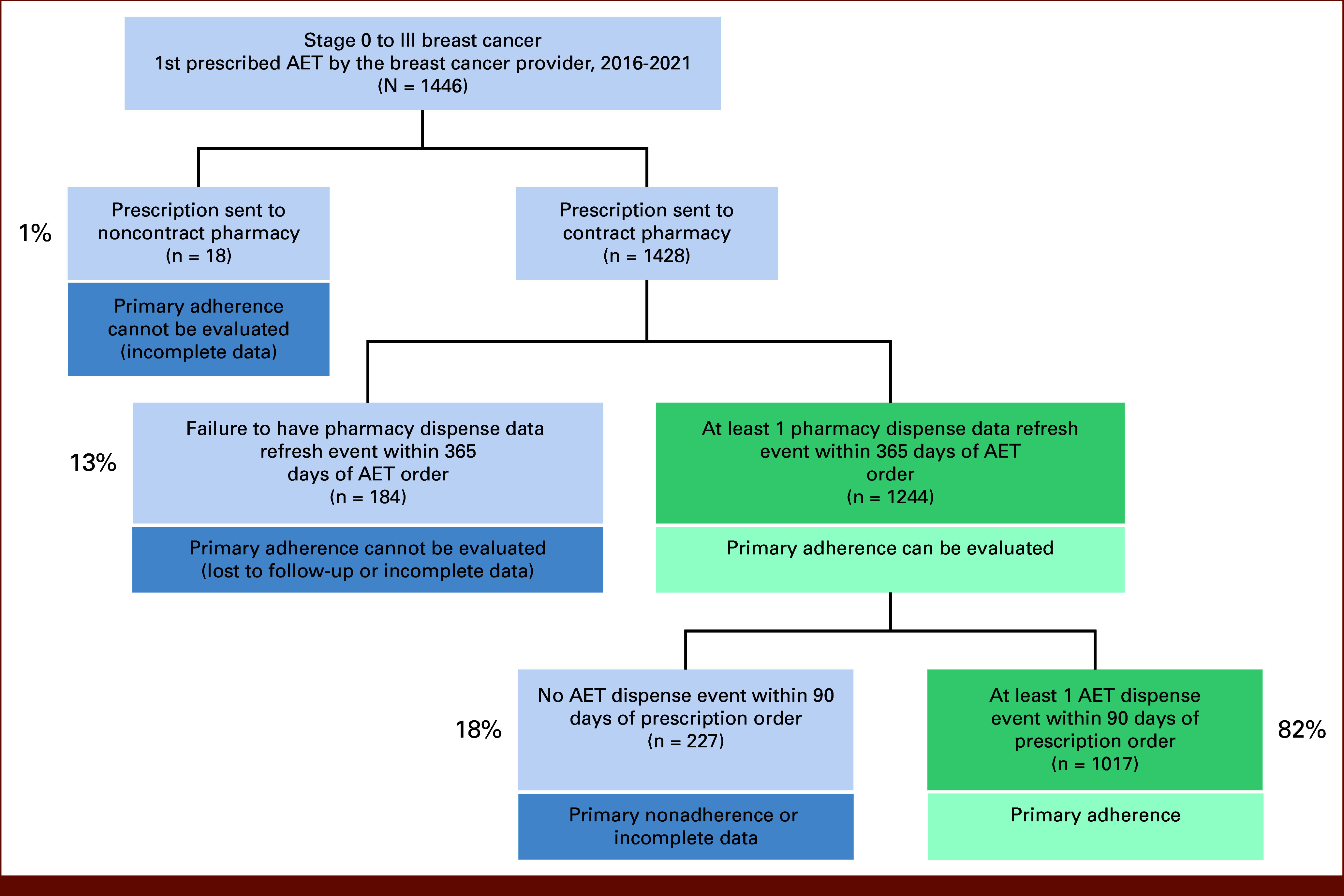
Patient selection pathway showing completeness and timeliness of linked EHR pharmacy data for early detection of primary adherence to AET. AET, adjuvant endocrine therapy; EHR, electronic health record.

From 2016 to 2021, 1,446 eligible patients with breast cancer had 1,446 first prescription orders for AET routed to 871 unique pharmacies. Of the 871 pharmacies, 856 (98%) were contract and 15 (1.8%) were noncontract. This resulted in 18 (1%) patients with incomplete data because their prescription was sent to a noncontract pharmacy. Among the remaining 1,428 (99%) patients who had their prescription sent to a contract pharmacy, 1,244 patients had at least 1 linked EHR pharmacy data refresh event within 365 days of the first AET order. The remaining 184 patients failed to have a refresh event in that time frame, corresponding to 13% for whom primary adherence could not be evaluated, likely because of lack of follow-up at the study institution. When combined with the patients whose prescription was sent to a noncontract pharmacy, this represents 14% of the study population with incomplete data. Among the remaining 86% (n = 1,244) of the population who could be evaluated for primary adherence, 1,017 (82%) patients had a documented first dispense within 90 days and were considered primarily adherent for AET (Fig 3). When viewed in aggregate, only 68% of the entire patient cohort had documented primary adherence according to the linked EHR pharmacy data. The remaining 32% of the patients had incomplete data (1%), lack of follow-up (13%), or lack of AET dispense within 90 days (18%).

### Timeliness of Receiving Data

**FIG 4.**
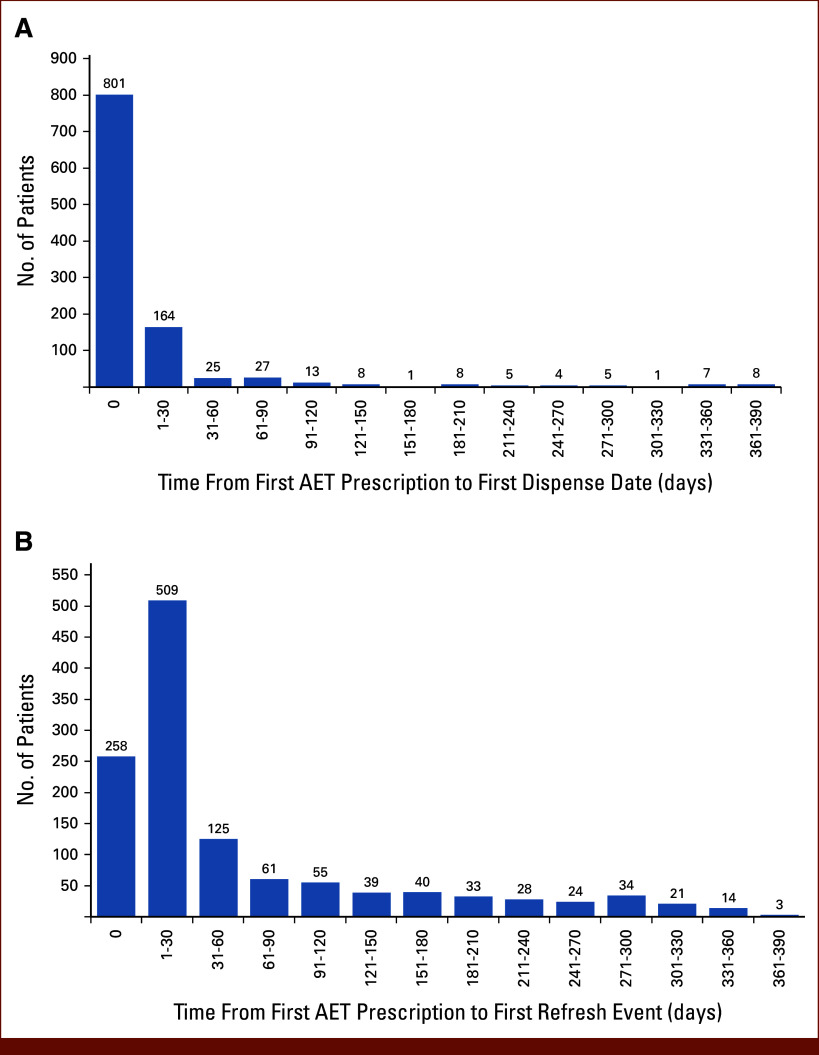
(A) Distribution of days between first AET prescription order and first dispense date; (B) distribution of days between first AET prescription order and first dispense refresh event date. AET, adjuvant endocrine therapy.

Among the 1,146 patients with at least 1 dispense event within 365 days of the first prescription order, 1,017 (89%) had at least 1 dispense within the first 90 days, making up the cohort of primary adherent patients (Fig 4A). Figure 4B shows 1,244 eligible patients for whom primary adherence can be evaluated. Of these, 970 (78%) had their data refreshed within the first 90 days. Table 2 combines the data from Figures 4A and 4B to illustrate that 953 (77%) patients had adequate data for early detection. The other 291 (23%) failed to have a refresh event within 90 days and were considered to have inadequate early detection. One hundred and eighty (18%) patients had order and dispensing events within the cutoff period but failed to have a refresh event within that period. These patients were primarily adherent but had inadequate data for early detection because the data were not refreshed in the system within 90 days of the prescription order.

**TABLE 2.** Distribution of Patients Who Have Adequate Data to Assess Primary Adherence

Category	Primary Nonadherence (n = 227), No. (%)	Primary Adherence (n = 1,017), No. (%)
Adequate early detection (n = 953, 77%)	116 (51)	837 (82)
Inadequate early detection (n = 291, 23%)	111 (49)	180 (18)

NOTE. The rows represent the distribution of patients who have a prescription order and refresh event within the cutoff period, making it possible to calculate adequate early detection and inadequate early detection. The columns represent the distribution of patients who have documented primary adherence and primary nonadherence.

## DISCUSSION

This study demonstrated the feasibility of using linked EHR pharmacy data for early detection of nonadherent patients with breast cancer who do not fill their first AET prescription within 90 days. The conclusion that this approach is feasible was based on evaluating how complete and timely the data were from a linked EHR pharmacy database. While the frequency of refreshing data could be improved to support more timely and complete data, linked EHR pharmacy data have the potential to improve clinician awareness of primary medication adherence.

Determining primary medication adherence is challenging since no single or standardized measure exists. Previous studies^12,13,20,21^ have evaluated adherence to AET in patients who fill at least one prescription, but do not evaluate the population at risk of never filling the prescription. These studies used various data sources to assess adherence, such as order, dispensing, or claims data but not linked EHR pharmacy data. Outside of oncology, a retrospective cohort study aimed to validate linked EHR pharmacy data to assess adherence of patients with an antihypertensive medication order. The results suggested that pharmacy fill data available in the EHR are sufficiently complete for assessment of medication adherence, but they did not assess the timeliness of the data for consideration of early interventions.^22^ Another study focusing on primary medication adherence evaluated a claims database to determine adherence to e-prescription data compiled over 1 year from the Massachusetts eRx Collaborative. A prescription was considered filled if there was a paid claim before the end of the 12-month study period. Seventy-two percent of e-prescriptions for new medications were filled.^23^ Although monitoring adherence with filled claims data is feasible, it is not useful for near real-time CDS to address primary adherence. The feasibility of using linked EHR pharmacy data, as demonstrated in this study, supports the possibility of achieving near–real-time CDS for AET primary adherence.

Different definitions of data completeness exist in the literature, and measurement of completeness depends on how triggering events are configured and how the data are received.^18,19,24^ Many institutions configured the triggering event for when a medication reconciliation should be performed when a clinical encounter was about to occur or was occurring. Each configuration directly affects the experience of completeness and timeliness of data. Incomplete refresh events pose an opportunity to enhance EHR systems to more frequently refresh pharmacy dispensing data for a specific population of interest. Linked EHR pharmacy data allow for other data refresh approaches, including time-based triggering events, which could be performed for all prescriptions, specific prescriptions, or specific patient populations. However, each transaction has a financial cost, which needs to be balanced with the value of performing an earlier triggering event. The creation of standardized approaches for triggering events could be an area of future research for the medical informatics community.

The primary study strengths include (1) a large, diverse sample collected over multiple years; (2) data obtained from multiple pharmacies through a linked EHR pharmacy database; and (3) primary adherence data collected from the first prescription order to the first dispense event.

There are some limitations to acknowledge. The study was conducted at a single institution in an urban setting, and it is not clear whether the results would generalize to other population settings. In addition, the impact of the variation in how triggering events are configured and how the data are received is difficult to assess as data refresh triggering rules can be specific to each institution. Similarly, the penetrance of the linked EHR pharmacy database in other markets could vary and affect the scope and quality of the data.

This study focused on primary nonadherence and did not assess persistence, in which patients stop taking medication after initiating it. The patterns and barriers to adherence and persistence may overlap but are not necessarily the same.^25^ Thus, future research would determine the ability of linked EHR pharmacy data to adequately assess persistence. The overall pattern of nonadherence could be used to assess factors associated with nonadherence and determine whether these differ for nonadherence and nonpersistence; differences may suggest interventions related to the type of nonadherence. Future work includes developing interventions in the EHR to notify the patient's care team of potential early nonadherence and nonpersistence and evaluating outcomes.

In conclusion, linked EHR pharmacy data represent a promising approach to provide CDS to breast cancer survivorship teams. We demonstrated this in the context of AET, which has the potential to increase the cure rate for breast cancer and yet has a high rate of primary nonadherence. Further improvement of the frequency and timeliness of data refreshing events has the potential to enhance linked EHR pharmacy data support of prospective CDS interventions to improve treatment outcomes.
